# Cell-Autonomous Sex Differences in Gene Expression in Chicken Bone Marrow–Derived Macrophages

**DOI:** 10.4049/jimmunol.1401982

**Published:** 2015-01-30

**Authors:** Carla Garcia-Morales, Sunil Nandi, Debiao Zhao, Kristin A. Sauter, Lonneke Vervelde, Derek McBride, Helen M. Sang, Mike Clinton, David A. Hume

**Affiliations:** Roslin Institute and Royal (Dick) School of Veterinary Studies, University of Edinburgh, Midlothian EH25 9RG, Scotland, United Kingdom

## Abstract

We have identified differences in gene expression in macrophages grown from the bone marrow of male and female chickens in recombinant chicken M-CSF (CSF1). Cells were profiled with or without treatment with bacterial LPS for 24 h. Approximately 600 transcripts were induced by prolonged LPS stimulation to an equal extent in the male and female macrophages. Many transcripts encoded on the Z chromosome were expressed ∼1.6-fold higher in males, reflecting a lack of dosage compensation in the homogametic sex. A smaller set of W chromosome–specific genes was expressed only in females. LPS signaling in mammals is associated with induction of type 1 IFN–responsive genes. Unexpectedly, because IFNs are encoded on the Z chromosome of chickens, unstimulated macrophages from the female birds expressed a set of known IFN-inducible genes at much higher levels than male cells under the same conditions. To confirm that these differences were not the consequence of the actions of gonadal hormones, we induced gonadal sex reversal to alter the hormonal environment of the developing chick and analyzed macrophages cultured from male, female, and female sex-reversed embryos. Gonadal sex reversal did not alter the sexually dimorphic expression of either sex-linked or IFN-responsive genes. We suggest that female birds compensate for the reduced dose of inducible IFN with a higher basal set point of IFN-responsive genes.

## Introduction

Gender-specific differences in innate and acquired immunity have been well-documented in mammals (1, 2). In general, these differences are attributed to direct and indirect actions of the sex hormones. For example, a recent study demonstrated that the female-specific bias in type 1 diabetes incidence in the NOD mouse was an indirect consequence of the effect of androgens on the gut microbiota (3). Others have demonstrated an interaction between estrogen and type 1 IFN signaling in regulating susceptibility in SLE models (4). Of course, males and females also differ in chromosomal complement. In mammals, the Y chromosome contains relatively few genes, and the double complement of genes on the X chromosome in females is controlled by X chromosome inactivation. It has generally been assumed that there are few Y chromosome–encoded functions other than testis determination; however, recent studies concluded that the Y chromosome genes encode proteins associated with general cellular functions (5, 6), and there is emerging evidence for effects of Y chromosome sequence polymorphisms on innate immunity and autoimmune pathology in both mice and humans (7). In birds, the heterogametic sex is female (ZW). Analysis of mixed-sex chimeric (gynandromorph) birds indicated that somatic cells in the chicken have an inherent cell-autonomous sex identity (8, 9). In a series of experiments where undifferentiated cells were transplanted between early chick embryos of different sexes, and the resulting chimeric embryos allowed to develop to an advanced stage, the transplanted donor cells retained their donor sex identity. Male donor cells expressed male-specific transcripts even when fully integrated into a female gonad, and vice versa (9). Although some Z chromosome genes appear to be expressed at similar levels in male and female birds (i.e., compensated) (10), the large majority are not, and dosage compensation in birds appears to be regulated on a gene-by-gene basis (11–13)

The type 1 IFN (IFN-α, IFN-β) clusters are located on the Z chromosome in birds (14). If IFN expression is not dosage compensated, one might anticipate that male birds would produce more effective defense against infections. However, the reverse appears to be the case. In a study involving a large number of chicken flocks (>300,000 male and female birds), mortality rates resulting from infectious diseases were ∼70% higher in males than in females (15). In this and other studies, the responses to bacterial, viral, and protein Ags were assayed, and females were found to both respond earlier and with higher Ab titers than males [e.g., to the major poultry pathogen Newcastle Disease virus (15, 16)]. IFN-β is a key regulator of antibacterial as well as antiviral responses in mammals. In murine macrophages responding to the TLR4 agonist, bacterial LPS, the transient induction of IFN-β via the MyD88-independent pathway and activation of transcription factors IRF1 and IRF3 leads to autocrine induction of IFN-responsive genes (17). LPS and IFN signaling is, in turn, subject to stringent feedback control by the inducible suppressor of cytokine signaling (SOCS1) (18, 19)

Studies of macrophage responses to LPS in mice and pigs have exploited the ability to grow macrophage cells from the marrow in M-CSF (CSF1) (20–22). This system also permits the identification of macrophage autonomous genetic differences in gene expression between strains and breeds, since the cells from different individuals are cultivated in a common culture environment ex vivo (23). We previously cloned and expressed chicken CSF1 and showed that it can be used to produce chicken bone marrow–derived macrophages (BMDM) (24). In the current study, we demonstrate that BMDM from male and female birds retain an intrinsic sex identity that includes differential expression of IFN-responsive autosomal genes.

## Materials and Methods

### Tissue preparation

ISA Brown eggs obtained from the National Avian Research Facility at the Roslin Institute were incubated in a humidified atmosphere at 39°C either to hatch, or for the required time. For fadrozole-induced gonadal sex reversal, the eggs were injected with either 1 mg fadrozole (Sigma-Aldrich) in PBS (10 mg/ml) or with PBS solution alone on day 3 of incubation (H&H Stage 18) and then reincubated for an additional 11 d for bone marrow collection. Embryos were then recovered and decapitated. Eggs were injected with the maximum tolerable dose of the aromatase inhibitor (1 mg/egg), and only treated embryos with gonads that had acquired a gross morphology that was fully male were used in our analysis. Gonad pairs were processed histologically and sections immunostained for the presence of male-specific and female-specific proteins. Abs were from AbD Serotec (aromatase) and from Millipore (SOX9).

Femurs and tibias were collected from sexed hatchling birds, or in the case of the sex reversal experiments, from embryos at day 14 of development and stored on ice while the sex of individual embryos was determined. Bones were stripped of muscle and bone marrow flushed using a fine needle and RPMI 1640 medium, under sterile conditions. Four separate pools of material, each from five individual birds of the same sex and treatment, were generated in each experiment. Bone marrow cells were cultured on bacteriological plastic dishes as previously described (24) in RPMI 1640 medium containing 10% FBS, 350 ng/ml recombinant chicken CSF1 (BMDM normal growth media) at 41°C for 1 wk to allow macrophage differentiation. At the end of this time, there is a confluent and pure population of macrophages. The macrophages were harvested by squirting them from the plate with medium, replated at a concentration of 10^6^ cells/ml in 6-well plates, and incubated with or without a predefined maximal dose of LPS [100 ng/ml, *Salmonella* Minnesota R595; Sigma-Aldrich; as used in previous studies in mammals (22, 23)] prior to RNA harvest.

### RNA extraction

Total RNA was extracted using RNA-Bee (AMS Biotechnology) according to the manufacturer’s instructions. RNA quality and quantity was assessed by Agilent Bioanalyser RNA 6000 Nanochip analysis and by Nanodrop spectroscopy (Thermo Scientific).

### Affymetrix array analysis

Microarray analysis was performed by ARK Genomics using Affymetrix systems and reagents. Hybridization probes were generated using the Ambion WT labeling kit and 500 ng RNA. Signal intensities were measured using a GeneChip Scanner 3000 7G and analyzed with GeneChip Command Console Software (AGCC), applying RMA normalization to the CEL files. To ensure robust male:female comparisons, only probes with signal intensities above the average of the lowest quartile + 3 SDs were included in our analyses. Microarray data have been deposited in the National Center for Biotechnology Information Gene Expression Omnibus (GSE59921) (http://www.ncbi.nlm.nih.gov/genbank).

### Functional enrichment analysis

Ensembl database entries were used to identify mammalian homologs for chicken genes and enrichment based on functional annotations determined by ToppFun analysis (ToppGene Suite; http://toppgene.cchmc.org/). The categories included in this analysis were: molecular function, biological process, cellular component, domain, pathway, interaction, and transcription factor binding site. The number of genes assigned to each category and the significance of the scores are shown. Only scores with *p* values ≤ 0.01 and representing a minimum of three genes are shown (assignments with a minimum of two genes are shown for the CASI-W analysis, because of the limited number of genes in this category).

## Results

### Expression profiling of male and female BMDM

BMDM were differentiated from bone marrow collected from 15 male birds and 15 female birds between 1 and 3 d after hatch. The bone marrow was obtained from newly hatched chicks to ensure that any differences were not due to gonadal hormones produced by sexually mature birds. Material from five individual birds was combined to reduce any impact of interindividual variation, and six pools representing three male and three female biological replicates were generated. Macrophages were harvested and then replated and cultured with or without the addition of LPS. The Affymetrix Chicken Genome Array was used to determine relative gene expression between male and female macrophages both before LPS treatment and 24 h after stimulation with LPS. The late time point was chosen to focus specifically on the downstream targets of endogenous IFN-β, which is a known early-response gene in macrophages.

**FIGURE 1.**
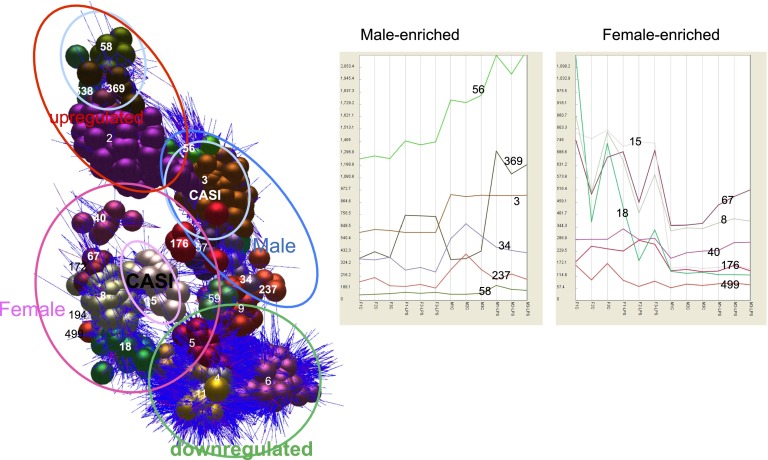
Network analysis of the gene expression profiles of male and female BMDM. Pools of bone marrow cells, each from five newly hatched male or female birds, were cultured in chCSF1 for 7 d, harvested, and cultured for an additional 24 h with or without LPS. mRNA was isolated and analyzed using Affymetrix expression arrays. The sets of coexpressed genes were identified using the network analysis tool Biolayout Express^3D^. The results are displayed in three dimensions, with nodes within each cluster given a common color, and each node joined by edges in blue. At *left*, the dataset clearly separates into four broad clusters, genes that are upregulated or downregulated by LPS to the same extent in each sex, and sets of genes that display female-, or male-enriched expression patterns. The clusters labeled cell autonomous sex identity (CASI) are largely encoded on the sex chromosomes. The panels at *right* show the average expression profiles of genes within distinct clusters showing male- or female-specific expression. From *left to right*, there are three control female samples (F1C, F2C, and F3C), then corresponding samples with LPS (F1-LPS, F2-LPS, and F3-LPS), three control males samples (M1-C, M2-C, and M3-C), and the corresponding samples treated with LPS (M1-LPS, M2-LPS, and M3-LPS).

**FIGURE 2.**
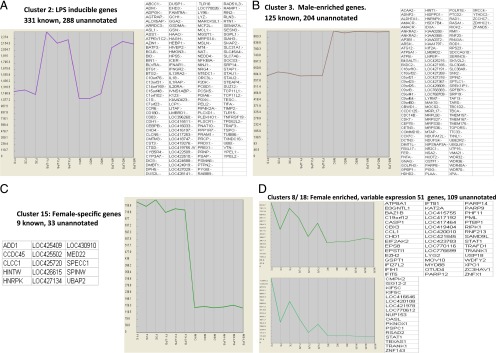
Gene expression profiles of clusters of coregulated genes in chicken BMDM. The figures show the average expression profiles of selected clusters of biological interest derived from the data in Fig. 1. From *left to right* in each panel, there are three control female samples (F1C, F2C, and F3C), then corresponding samples with LPS (F1-LPS, F2-LPS, and F3-LPS), three control males samples (M1-C, M2-C, and M3-C), and the corresponding samples treated with LPS (M1-LPS, M2-LPS, and M3-LPS). In each panel, the box at *right* lists the annotated transcripts within each cluster.

To analyze the data, we initially used the network-based analytical tool, Biolayout Express^3D^ (25). As shown in Fig. 1A, the dataset could be segregated into a substantial number of clusters of genes sharing similar patterns of transcription. Each of the clusters can be represented as an average expression profile of its members (Fig. 1B), highlighting patterns of particular interest. Four of the most informative clusters are shown in detail in Fig. 2. Fig. 2A shows one of the largest clusters, cluster 2, which contains some 600 probe sets. The genes within this cluster were equally expressed in macrophages from male and female birds, and induced to a similar extent in all three pooled replicates by 24-h LPS stimulation. It contains the very well studied lysozyme gene, which is known to be profoundly LPS-inducible in the chicken (26), and many other transcripts describe previously in a study of induction of gene expression in HD11 macrophage line by LPS (27). As noted in previous analyses of the responses of mouse, pig, and human macrophages to LPS (20–22), this late-responsive cluster includes many known feedback regulators of TLR4 signaling, such as *Batf3*, *Dusp1*, and *Nfkbia.* As in mammals, the LPS-induced cluster does contain both the type 1 (*Ifnar1*) and type II (*Ifngr2*) IFNRs. However, the known IFN-induced genes are absent from this set of LPS-induced genes, suggesting either that the MyD88-independent pathway was not activated in chicken BMDM, or that the two sexes differ in their response. One obvious possibility was that these genes would be more highly induced in the male cells because of the presence of the IFN clusters on the Z chromosome, but the only cluster conforming to that pattern, cluster 369, contained only three genes. Of these, only the chemokine gene annotated as IL8 (CXCL1; ∼2-fold more inducible in males, from a similar basal level to females) was highly expressed and has a known function.

Cluster 3 contains ∼330 clone sets, and the average expression profile displays ∼1.5-fold enrichment in all the male-derived samples (Fig. 2B). Many of these transcripts are encoded on the Z chromosome, summarized in detail in Supplemental Table IA. The data confirm the significance of the differential expression, and the tight clustering of relative expression in macrophages from males at an average of 1.63-fold enrichment compared with the female. A corresponding small cluster of transcripts (cluster 15; Fig. 2C) derived from the W chromosome was detected only in macrophages from female birds. None of these differentially expressed sex chromosome encoded transcripts in either males or females has a known function in the regulation of innate immune function.

**FIGURE 3.**
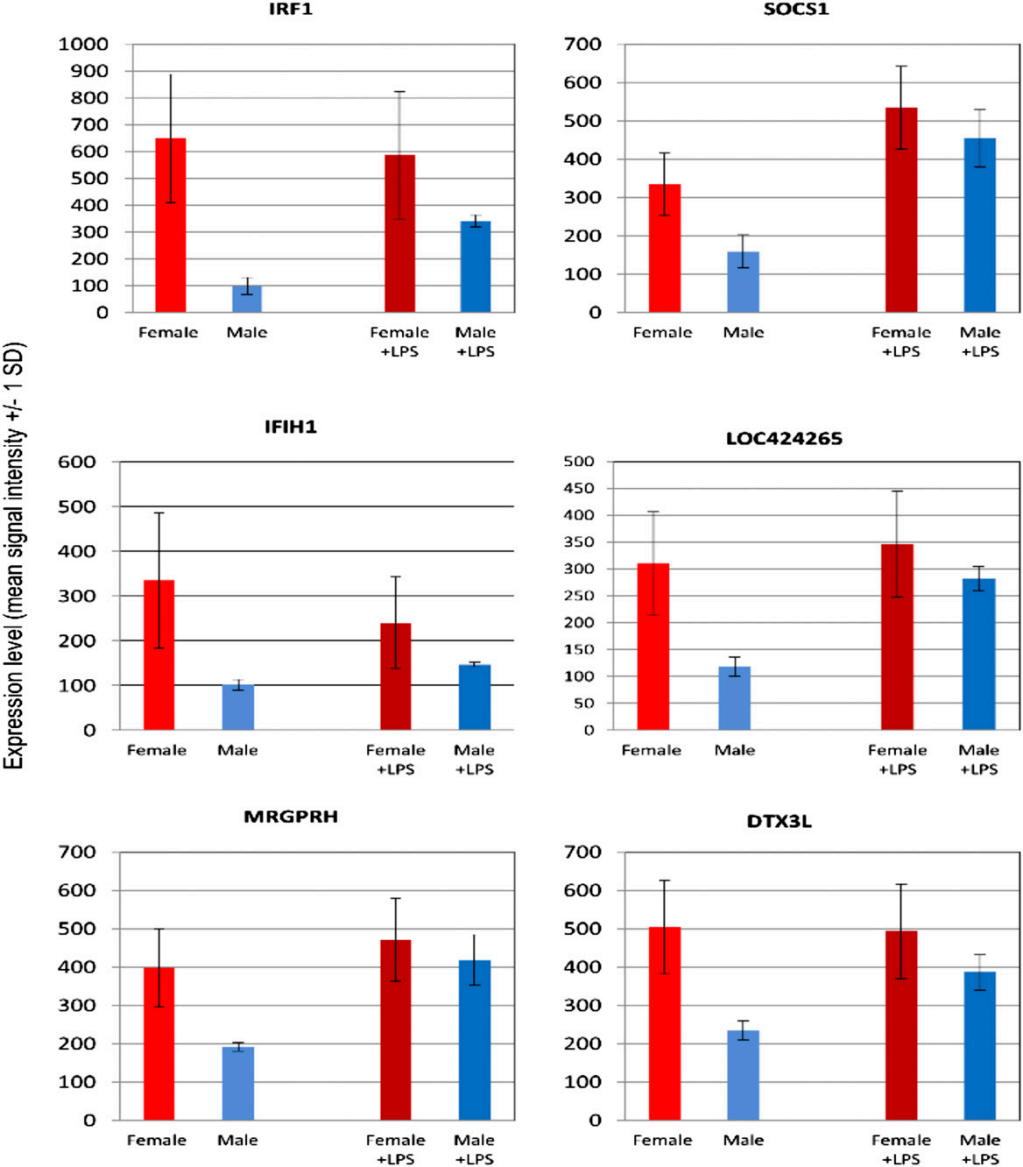
Gene expression profiles of individual LPS-responsive genes in BMDM from male and female birds. Representative expression profiles of a subset of genes expressed constitutively at higher levels in macrophages from female birds and induced in male birds. The results are the average of three separate pools. The primary data, additional annotation and other genes with this profile are provided in Supplemental Table ID.

The reason why known IFN-inducible genes are absent from the common LPS-induced cluster (cluster 2) is that they formed two separate clusters (clusters 8 and 18; Fig. 2D), in which the expression was higher in macrophages from female than male birds, and was also significantly different between the individual pools from females. The expression pattern in females has a dominant influence on the correlation coefficient that underlies the clustering method, and obscured the fact that many of these genes are, in fact, LPS-inducible in males. Supplemental Table IB summarizes the data for all of the genes that were significantly more highly expressed in the macrophages from females, and Supplemental Table IC summarizes a GO analysis, highlighting the clear enrichment in known IFN-responsive transcripts. Fig. 3 shows the small subset of genes within this set that is inducible by LPS in the BMDM from males to the level seen constitutively in females (see also more detailed descriptions in Supplemental Table ID). A notable member of this set is the inducible feedback regulator of IFN signaling, SOCS1 (18, 19).

To summarize, there is a large set of transcripts that distinguishes cells from male and female birds, most of which are associated with the sex chromosomes, and the macrophages of female birds express IFN-responsive genes constitutively.

### Effect of sex-reversal on sexually dimorphic expression

**FIGURE 4.**
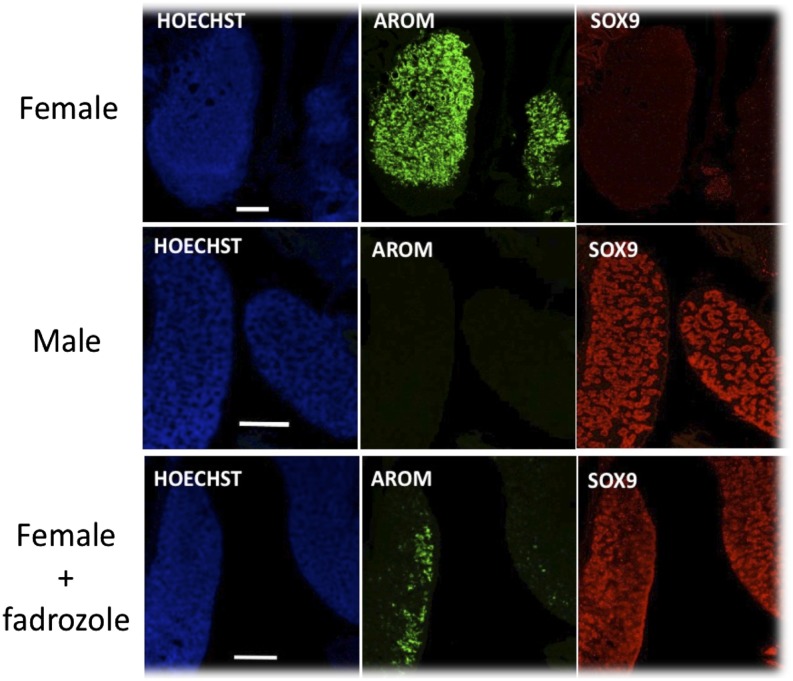
Effect of fadrozole on expression of sex-specific proteins in the chick embryo gonads. Panels show images generated from histological sections through the left and right gonads of a PBS-treated female embryo, the left and right gonads of a PBS-treated male embryo, and the left and right gonads of a fadrozole-treated female embryo. Images show each section stained with the nuclear dye, Hoechst (blue), and immunostained for expression of Aromatase (green) and SOX9 (red). Fadrozole-treated female gonads clearly adopt a testicular morphology and show reduced levels of aromatase and obvious expression of the male-specific SOX9. Scale bars, 20 μm.

Despite the common tissue culture environment and the extensive proliferation of progenitors that occurs in the cell culture used to generate BMDM, there is a formal possibility that differential gene expression is an epigenetic consequence of the organizational effects of male and female embryonic hormones. To eliminate this possibility, we produced sex-reversed birds. Sex reversal of the ZW gonad was achieved by treatment with an inhibitor of aromatase enzyme activity (fadrozole), which prevents the production of estrogen from androgens and produces gonadal sex reversal in female birds (28). Eggs were injected with either fadrozole ([1 mg/egg] or with PBS on day 3 of incubation [H&H Stage 18]) and then reincubated for an additional 11 d to collect bone marrow. Gonads from genetic female embryos treated with fadrozole expressed the testis-associated marker Sox9, showed reduced levels of the aromatase enzyme, and acquired the gross morphology and internal structures (sex cords) typical of a developing testis (Fig. 4). The Affymetrix Chicken Gene 1.0 ST Array was used to monitor gene expression in macrophages from male, female, and sex-reversed female embryos. In effect, this is a complete replication of the analysis, albeit using marrow from an earlier developmental stage. The complete datasets are provided in Supplemental Tables II and III. Fadrozole had no significant effect on the expression of the Z chromosome genes, which continued to be expressed in a sexually dimorphic fashion in male and female macrophages derived from sex-reversed embryos (Supplemental Table IIIB). Crucially, the female-biased expression of IFN-target genes was not suppressed (masculinized) by altering the embryonic hormonal environment from female to male (Supplemental Table IIIC).

## Discussion

Although there have been a number of reports on the transcriptomic response of chicken macrophage cell lines to LPS (27), to our knowledge, this is the first study of primary chicken BMDM that illustrates their use as a system. In mouse (21) and pig (23), analysis of BMDM provided a system in which cells from different individuals or strains can be assayed in a common culture system, removed from effects of other cell types or environment. The response to LPS in macrophages is a complex transcriptional cascade (21, 29–31), starting with immediate early genes transcribed from poised RNA-pol II complexes and followed by induction of downstream targets by the early gene products (32). Those products include both inducible transcription factors and autocrine regulators including the many proinflammatory cytokines.

At the same time, there is the induction of a large cohort of feedback regulators, so-called inflammation suppressor genes (33), which will eventually damp the inflammatory response and return the cells by 18–24 h to a new steady state that is resistant to further stimulation by LPS. In the current study, we focused specifically on that later 24-h time point. By that time, we noted the induced expression of some 600 transcripts that were shared by macrophages from males and females, including many of the known feedback regulators. No doubt there are many more such regulators among the large set of genes on the array platform that await informative annotation.

A recent eQTL analysis of the response of human peripheral blood monocytes to LPS identified a cohort of genes induced at 24 h that was correlated in trans with sequence variation at the IFN-β locus (34). In mammalian (18, 21) and avian (27) macrophages, IFN-β is an immediate early gene and induced transiently by LPS; we anticipated that measurement of the downstream target genes would provide a more sensitive indication of any differential regulation of the pathway between male and female macrophages as a consequence of the presence of the IFN cluster on the Z chromosome. The pattern we observed was the direct reverse of the anticipated outcome; the macrophages from the female birds expressed many known IFN-responsive genes constitutively, associated with relatively higher expression of the IFN-responsive transcription factors IRF1 and IRF7 (Supplemental Table IB). We were not able to detect IFN-β mRNA by quantitative RT-PCR in either male or female cells (data not shown) or on the arrays. However, like the response to LPS, the response to exogenous type 1 IFN in mouse macrophages is transient and is subject to inducible feedback control, notably by SOCS1 (18, 21). Interestingly, commonly used mouse strains, C57BL/6 and BALB/c also differ in the basal expression of many known IFN-responsive genes, an observation that has been attributed to low level constitutive expression of IFN-β in the C57BL/6 line (21). Accordingly, we suggest that the underlying mechanism is that BMDM of the chicken, like those of the mouse, produce endogenous IFN at low levels. In males, with the entire cluster of type 1 IFN genes on the Z chromosome, the level of expression induces both a larger initial response and a more complete shutdown. In females, the lower expression of IFN also produces a less-effective induction of feedback regulators, and the overall induction of IFN target genes is sustained. However, it is also possible that other Z chromosome– or W chromosome–encoded transcripts contribute to the shutdown of IFN-responsive genes in males or their sustained induction in females.

We can only infer that the differential expression of the IFN-responsive genes in females underlies their greater resistance to pathogens. Most studies of avian infectious disease susceptibility, including studies of pathogen-induced gene expression use mixed populations of males and females and made no distinctions between their responses. Similarly, we do not have direct evidence that the differences in expression of IFN-responsive gene expression are due to lack of dosage compensation on the Z chromosome. The original study that localized the genes to the Z chromosome addressed this question but could not demonstrate differences in IFN production between the sexes because of a high degree of variation in virus-induced expression between individuals of both sexes (35). Interestingly, even though we examined pools of macrophages from five birds, there was substantial variation between the pools in expression of the IFN-inducible genes as a class (Fig. 2D), which is likely to be associated with variation in the common regulator, IFN. Genes on the Z chromosome have evolved more rapidly than the autosomes in avian species (36). Because functional variation in the single copy of the IFN loci in the female line would immediately impact upon antiviral defense and the vast majority of viruses have evolved mechanism to evade or compromise IFN-mediated defenses (37), one would expect the IFN genes to be especially subject to selection. Chicken BMDM cultured as described herein can be infected with several viral pathogens including Marek’s disease virus and infectious bronchitis virus (IBV). However, despite the basla differences in expression of IFN-responsive genes, we did not observe any difference in the replication of infectious bronchitis virus when macrophages from male and female birds were compared (K.A. Sauter and L. Vervelde, unpublished observations). The BMDM system could be used to assess the impact of genetic variation on the response to viral or bacterial pathogen challenge, or the effect of genetic background as we have done previously in the pig (23). Clearly, such studies in birds would need to examine the responses of male and female birds separately.

The use of sex-reversed birds demonstrated clearly that the sexually dimorphic characteristics are inherent features of male and female macrophages and unrelated to prior gonadal hormone exposure. Aside from the IFN genes, the male and female macrophages differed from each other in their expression of many other genes on the Z and W chromosomes. The majority of these genes are also differentially expressed in other embryonic and adult tissues and seem to be the hallmarks of cell-autonomous sex identity (D. Zhao, D. McBride, S. Nandi, and M. Clinton, manuscript in preparation). Our findings would support the proposal by Mank and Ellegren (13) that the limited sex chromosome dosage compensation in birds occurs on a gene-by-gene, rather than a chromosome-wide basis, as appears to be the case for random allelic inactivation on the autosomes in mammals (38). None of the genes overexpressed in a male-specific manner has a known immune function, but the female-specific W chromosome–associated genes (Supplemental Table IIB) are significantly enriched for genes that interact with IFN-stimulated gene 15, a ubiquitin-like protein that is conjugated to intracellular target proteins upon IFN activation (39). So, like the mammalian Y chromosome, the avian W could potentially have genes that contribute to immune regulation and certainly does contribute to a novel female macrophage-associated transcriptome.

## Supplementary Material

Data Supplement
