# Supplementary material for: Cell-Autonomous Sex Differences in Gene Expression in Chicken Bone Marrow–Derived Macrophages
Source: J Immunol. 2015 Jan 30;194(5):2338–44. doi: 10.4049/jimmunol.1401982 (PMC4337484; doi:10.4049/jimmunol.1401982)
Supplement: Data Supplement [file JI_1401982.zip › JI_1401982_Supplemental_Material_1.pdf]

Legends to Supplementary Tables

Tables S1

Macrophages were differentiated from bone marrow collected from 15 male birds and 15 female birds between 1 and 3 days after hatch. Material from five individual birds was combined and six pools representing three male and three female biological replicates were generated. Cells isolated from bone marrow were cultured in the presence of recombinant chicken CSF-1 for seven days. Microarray (Affymetrix Chicken Genome Array) analysis of gene expression in pools of female (F) or male (M) macrophages grown in standard conditions (controls = C) or in the presence of lipopolysaccharide (treated = LPS).

Genes are identified by information in the first three columns of table: Gene Symbol, Representative Public ID and Probe Set ID. Table shows signal intensity for three pools of female macrophages (Female Control 1-3), three pools of LPS-treated female macrophages (Female +LPS 1-3), three pools of male macrophages (Male Control 1-3), and three pools of LPS-treated male macrophages (Male +LPS 1-3). Table shows mean values for male and female samples and for LPS-treated male and female samples. Male:female ratios are calculated for Control and LPS-treated samples.

Table S1 provides a separate analysis of genes encoded by the sex chromosomes (S1A) and autosomal genes expressed more highly in macrophages from females than males (S1B) and the functional enrichments associated with the set of female-specific autosomal genes (S1C). Table S1D summarises the subset of those genes that are induced by LPS in the male-derived cells.

Table S2 and Table S3.

a. Expression in macrophages derived from PBS-treated and fadrozole-treated male and female chick embryos.

At day 4 of development, ISA Brown eggs were injected with either 1mg fadrozole (Sigma) in phosphate buffered saline (PBS) (10mg/ml), or with PBS solution alone. Eggs were sealed with tape and re-incubated until day 14 of development and then embryos recovered. Bone marrow-derived

macrophages were generated by culture in CSF1 and subjected to expression profiling. Genes are identified by information in the first four columns of table: Affymetrix Probe set ID, Gene Symbol, Start site and mRNA Accession number. The entire dataset is contained in Table S3. The Table shows the signal intensity for four pools of macrophages from male embryos, four pools of macrophages from female embryos, four pools of macrophages from fadrozole-treated male embryos and four pools of macrophages from fadrozole-treated female embryos.

Table S2 highlights the subset of genes specifically distinguish the male and female birds. S2A examines the differential expression of genes encoded on the sex chromosomes. S2B tabulates the expression of sexually dimorphic interferon-responsive transcripts in macrophages from sex-reversed embryos. Expression levels were compared by Student T-test (p-value).
